# Genetic Screening of Frontotemporal Dementia Patients from a Memory Clinic in Argentina

**DOI:** 10.1002/alz.085944

**Published:** 2025-01-03

**Authors:** Ana Laura Martinez Ojeda, Nahuel Magrath Guimet, Pablo Miguel Bagnati, Ricardo Allegri, Jorge Campos, Gustavo Sevlever, Ezequiel Ignacio Surace

**Affiliations:** ^1^ FLENI INEU‐CONICET, Buenos Aires, Buenos Aires Argentina; ^2^ Fleni, Buenos Aires Argentina; ^3^ Fleni, Buenos Aires, CABA Argentina; ^4^ FLENI, CABA Argentina; ^5^ Fleni, Buenos Aires, Buenos Aires Argentina

## Abstract

**Background:**

Frontotemporal dementia (FTD) remains one of the most common forms of early‐onset dementia (45 to 65 years). FTD consists clinically and pathologically of a heterogeneous group of disorders characterized by progressive frontal and temporal lobe atrophy. Thirty to fifty percent of cases have a family history of the disease. In this work we aimed to characterize pathogenic variants implicated in familial FTD cases in a cohort from Argentina.

**Method:**

A total of 43 patients (23 women, 20 men) meeting clinical criteria for probable behavioral variant‐FTD, semantic or nonfluent primary progressive aphasia, according to the Rascovsky and Gorno‐Tempini criteria, were evaluated at a memory clinic in Buenos Aires, Argentina. Genomic DNA was obtained from peripheral blood leukocytes for all patients using standard protocols. *C9ORF72* hexanucleotide repeat expansion (C9‐HRE) was assessed by repeat‐primed PCR. Whole exome sequencing and bioinformatic analysis of FTD‐causative genes was performed on C9‐HRE negative cases.

**Result:**

29 out of 43 patients (67%) were familial cases, of which 3 were C9‐HRE positive (10.3%). Eleven familial cases who were C9‐HRE negative were studied by exome sequencing. We found previously reported pathogenic variants in 2 cases [*GRN* (NM_002087.4): c.1216C>T: p.Gln406Ter and *GRN* (NM_002087.4): c.709‐1G>A: p.?], respectively. Also, we found two variants of unknown significance in other two probands [*PDE11A* (NM_016953.4): c.2518G>A: p.Glu840Lys; *PSEN2* (NM_000447.3): c.800T>G: p.Val267Gly], respectively.

**Conclusion:**

Here we report the genetic screening of familial FTD cases from a memory clinic in Argentina. C9‐HRE is still the most common cause of familial FTD. Further studies are warranted on the role of the *PSEN2* and *PDE11A* variants found in two probands. This work adds to the knowledge of the genetic landscape of FTD in our country.

